# Threshold optimization in separating cortical and extracerebral hemodynamics using principal component analysis

**DOI:** 10.3389/fnhum.2026.1778201

**Published:** 2026-04-22

**Authors:** Wakana Kawai, Kazuki Hyodo, Yuki Yamamoto, Tatsuya Hayashi, Daisuke Yamaguchi, Aiko Ueno, Simone Cutini, Ippeita Dan

**Affiliations:** 1 Applied Cognitive Neuroscience Laboratory, Chuo University, Tokyo, Japan; 2 Meiji Yasuda Life Foundation of Health and Welfare, Physical Fitness Research Institute, Tokyo, Japan; 3 Faculty of Science and Engineering, Yamato University, Osaka, Japan; 4 Department of Developmental Psychology, Padova, Italy

**Keywords:** functional near-infrared spectroscopy (fNIRS), n-back task, principal component analysis (PCA), short-separation channel, threshold optimization

## Abstract

**Introduction:**

When trying to differentiate between hemodynamic cortical and extracerebral signals identified by devices used to detect cortical activity, statistical methods such as principal component analysis (PCA) are commonly employed as alternative approaches to using short separation measurements to reduce the influence of extracerebral hemodynamics. PCA requires a threshold value to separate cortical and extracerebral signals; however, existing methods often rely on fixed thresholds that fail to account for inter-individual variability and differences in experimental design, potentially leading to over- or under-correction. Rather than introducing a novel extracerebral hemodynamics removal method, the present study aims to optimize the use of existing methodologies. Specifically, we proposed a method to optimize the threshold that differentiates cortical from extracerebral hemodynamics in PCA-based analyses.

**Methods:**

Each of the four analyses were applied to a dataset obtained from older participants performing a verbal n-back task: (1) no correction (NC), (2) short separation regression (SSR), (3) PCA with our proposed threshold optimization (PCA_opt_), and (4) PCA with the individual maximum as threshold (PCA_max_). Bayesian *t*-tests were then conducted to evaluate the equivalence between SSR and PCA_opt_.

**Results:**

NC displayed the strongest cortical activation, PCA_max_ the weakest. SSR and PCA_opt_ produced intermediate results, and Bayesian t-tests revealed that the BF_01_ values for most of the channels were greater than 3.0, whereas no channels exhibited corresponding BF_10_ values exceeding 3.0.

**Discussion:**

Optimizing the threshold for separating cortical and extracerebral hemodynamics is a practical and effective strategy when using PCA as an alternative to short-separation measurements. This approach enables appropriate correction even in the absence of short-separation channels.

## 1 Introduction

Functional near-infrared spectroscopy (fNIRS) is a non-invasive brain imaging technique that measures hemodynamic signals corresponding to relative concentrations of oxygenated (oxy-) and deoxygenated hemoglobin (deoxy-Hb), reflecting cortical activation (Jöbsis, 1977; Villringer et al., 1993). fNIRS is employed to assess cortical activation across various populations and research domains owing to its robustness to motion, ease of use, portability, and applicability even in naturalistic environments (Ferrari and Quaresima, 2012; Pinti et al., 2020). Nonetheless, fNIRS signals are inevitably contaminated by extracerebral hemodynamics (Kirilina et al., 2012; Nasseri et al., 2018; Tong et al., 2011). This occurs because emitters transmit near-infrared light that passes through the extracranial tissues, skull, and cerebral cortex, and detectors capture the light, which is reflected back through the same layers in reverse order. Contamination by extracerebral hemodynamics is problematic, as it can lead to false-positive or false-negative detections of cortical activation (Tachtsidis and Scholkmann, 2016). Therefore, appropriate measures must be taken to mitigate its effects (Yücel et al., 2021).

One way to remove extracerebral hemodynamic contamination is to adopt short-separation (SS) channels (CHs) in addition to long-separation (LS) CHs. A SS-CH is defined as a channel with an emitter–detector distance of less than 15 mm (Brigadoi and Cooper, 2015), whereas conventional LS-CHs typically have a distance of approximately 30 mm. As SS-CHs have a shorter emitter–detector distance than LS-CHs, the emitted light does not reach the cerebral cortex as it does with LS-CHs. Therefore, signals measured by SS-CHs are assumed to reflect only the noise components, which is to say extracerebral activity. By utilizing the data from SS-CHs, such as adding it as a regressor in a general linear model (GLM) analysis, hemodynamic effects of the extracerebral areas could be mitigated, and relevant cortical signals could be better estimated than without it (Saager and Berger, 2005, 2008). Utilizing SS-CHs is said to be the most effective method for removing extracerebral hemodynamic data from continuous-wave fNIRS data (Santosa et al., 2020; Yücel et al., 2021). Nevertheless, this method is not without limitations. First, it cannot be applied to previously collected datasets that lack SS-CH measurements. Additionally, some instruments still do not support measurement using SS-CHs. Therefore, alternative methods that can remove extracerebral hemodynamic data without relying on SS-CHs could be a significantly advantageous, ensuring applicability across a wide range of contexts.

Common approaches for removing extracerebral hemodynamic data without SS-CHs involve decomposing measured fNIRS signals into cortical and extracerebral components using statistical methods such as principal component analysis (PCA) (Zhang et al., 2005, 2016) and independent component analysis (ICA) (Kohno et al., 2007). For example, in a PCA-based approach (Zhang et al., 2005) common components between task and resting-state data were extracted using PCA and removed as they were assumed to reflect systemic physiological signals. A drawback of this method, however, is that it requires additional resting-state data. As an alternative, another PCA-based approach, which utilizes the spatial distances between channels derived from their three-dimensional coordinates without the need for resting-state data, has been proposed (Zhang et al., 2016, 2017). In Noah et al., the components removed by this alternative approach were shown to resemble the signals measured by SS-CHs (Noah et al., 2021). Given that SS-CH signals are assumed to primarily reflect extracerebral hemodynamics, the authors concluded that their proposed method effectively reduced the influence of extracerebral components.

As an ICA-based approach, Kohno et al. propose that the spatially uniform components be removed from the signals decomposed broken down by ICA. The components removed using their proposed method showed a high correlation with signals measured using Doppler flowmetry on the forehead. Since Doppler flowmetry on the forehead primarily reflects scalp hemodynamics—the main component of extracerebral hemodynamics—they concluded that their method effectively removes scalp hemodynamics, thus reducing the effect of extracerebral hemodynamics. These methods enable the reduction, but not removal, of the effects of extracerebral hemodynamics even in the absence of SS-CH measurements.

Although PCA- and ICA-based approaches can effectively reduce the influence of extracerebral hemodynamics, they are not without limitations. They require a parameter or threshold to distinguish cerebral from extracerebral signals. However, the criterion for setting this value is often either ambiguous or fixed and is determined without considering differences in experimental tasks or regions of interest (ROIs). For instance, in the PCA-based resting-state-data filter method described above (Zhang et al., 2005), a threshold was required to identify components corresponding to global systemic effects within the PCA-decomposed resting-state signal. In Zhang et al.’s study the maximum eigenvalue was used as the threshold, whereas in another study utilizing their method, a threshold based on 80% of the variance, which corresponded to the first and second largest eigenvalues, was adopted (Franceschini et al., 2006). Additionally, in the PCA-based spatial-information filter method described above (Zhang et al., 2016), a predefined parameter was required. Initially, it was set to a value which was estimated to be broader than the expected cortical activation and narrower than the extent of scalp hemodynamics. This value was later adjusted based on results from a tapping task, in which activation in the motor cortex peaked, and the same filter width was ultimately applied to an overt-speaking task (Zhang et al., 2017). Finally, in the ICA-based approach described above (Kohno et al., 2007), a new statistical metric called the coefficient of spatial uniformity (CSU) was introduced. This metric was used to identify components representing spatially uniform signals, as scalp hemodynamics were assumed to be homogeneously distributed across all the measurement channels. A threshold was required to exclude noise components, and components with CSU values exceeding this threshold were removed. In their study, Kohno et al. used the maximum CSU for each individual as the threshold. Similarly, some studies have used the maximum CSU value for each individual as the threshold (Virtanen et al., 2009; Zhang et al., 2021), while others have applied thresholds such as one-sixth of the maximum (Wang et al., 2018) or a fixed value of 1.5 (Bae et al., 2020). In summary, regardless of whether PCA- or ICA-based approaches are used, each method relies on a parameter or threshold to distinguish between cerebral and extracerebral signals. However, the specific threshold values vary across studies, even among those employing the same filtering method or analytical approach.

One way to address the variability in threshold values is to determine an optimal fixed value for each approach, as suggested in some studies (Zhang et al., 2016, 2017, 2021). However, variation in threshold values across studies may also be reasonable, as it could be reflecting differences in experimental design, experimental tasks, or participant characteristics. For example, decreases in skin blood flow have been observed during arithmetic and stress-inducing tasks (Kirilina et al., 2012), whereas increases in skin blood flow and blood volume have been reported in studies employing word fluency tasks (Takahashi et al., 2011). Moreover, individual differences may also impact the size of scalp hemodynamics. For example, using the ICA-based approach described above (Kohno et al., 2007), one study reported maximum CSU values of 2.82 and 2.74 for individual participants (Kohno et al., 2007), whereas another study reported a mean maximum CSU across participants of 1.74 ± 0.77 (Zhang et al., 2021), which is substantially lower than the former. In addition, another study reported that their results were influenced by variations in trials, instruments, and participants, despite using the same overt speaking task (Nasseri et al., 2018). These findings also suggest that using each participant’s individual maximum value (Kohno et al., 2007; Virtanen et al., 2009; Zhang et al., 2021), rather than a fixed threshold, may still result in sufficient correction for some participants but be insufficient correction for others. Taken together, adjusting the threshold values for each study may be a reasonable approach to account for differences in experimental designs, tasks, or participant characteristics. However, allowing thresholds to vary across studies introduces another issue, as it may lead to subjective or arbitrary threshold settings, potentially resulting in false-positive or false-negative detection of cortical activation. To avoid subjectively determining threshold values for each study, it is necessary to develop a method for optimizing the threshold in a study-specific manner.

In the present study, we proposed a method to optimize the threshold to separate cerebral and extracerebral hemodynamics. This threshold optimization method enables an objective, study-specific optimization of the threshold, in contrast to the conventional subjective and predefined threshold. Importantly, it does not require additional equipment such as SS-CHs or supplementary data such as resting-state recordings. Rather than introducing a novel extracerebral hemodynamics removal method, the present study aims to optimize the use of existing methodologies. Specifically, we focused on using PCA, with CSU (Kohno et al., 2007) as an index, and proposed a threshold optimization method adapted for use within this framework. While this framework was used in the present study, the principle of the proposed method, removing components exhibiting the highest uniformity CHs, is not inherently limited to this method and may be extended to other approaches.

In essence, this threshold optimization method consists of two steps following the decomposition of fNIRS signals using PCA: (1) defining the threshold exploration range and (2) selecting the optimal threshold. CSU (Kohno et al., 2007) was used as an index to evaluate the spatial uniformity of components. Subsequently, we evaluated whether applying PCA with the proposed threshold optimization method could effectively reduce the influence of extracerebral hemodynamics. We used a dataset obtained from older participants performing a verbal n-back task, a commonly used cognitive task. Since the bilateral prefrontal cortex (PFC) was significantly activated during a 2-back task in a previous study (Hyodo et al., 2024), significant activation was expected in CHs located in those regions. To demonstrate the effectiveness of our proposed threshold optimization method, we applied four analyses to the data: (1) No Correction (NC), (2) Short Separation Regression (SSR), (3) PCA with threshold optimization (PCA_opt_), and (4) PCA with the individual maximum threshold (PCA_max_). Since NC analysis does not account for extracerebral hemodynamics, it was expected to be more prone to false positives and to detect greater activation compared to the other three analysis methods. Also, compared to the conventional approach of using individual maximums (PCA_max_), PCA_opt_ was expected to yield activation patterns more similar to those obtained with SSR, as its thresholds were optimized based on the current dataset. If the proposed threshold optimization method effectively reduced the influence of extracerebral hemodynamics, it would be expected to yield activation patterns similar to SSR. Therefore, we anticipated that PCA_opt_ and SSR would produce comparable activation results and we conducted a Bayesian *t*-test to confirm that there was no significant difference in the magnitude of activation detected.

## 2 Materials and methods

### 2.1 Dataset

We utilized baseline verbal n-back data from an exercise intervention trial conducted in community-dwelling older adults (UMIN-CTR: UMIN000044758). A total of 101 participants attended the eligibility assessment and baseline session. Twenty-three of the participants met the exclusion criteria and were excluded from the analysis. Specifically, five participants had a psychiatric disorder or were taking psychotropic medication, seven had either a history of neurological disease or suspected dementia as indicated by the Mini-Mental State Examination–Japanese (MMSE-J; score < 23), three were unable to exercise, which was essential for the original purpose of this dataset acquisition, three were left-handed, and five withdrew from the study. In addition, 15 participants were excluded based on issues identified during the measurement process. Specifically, two had measurement errors, three declined to undergo the n-back task, and ten performed below chance level in the n-back task. Ultimately, data from 63 participants was used in the analysis (mean age: 75.2 ± 5.27 years; 9 males and 54 females). The experiment was approved by the ethics committee of Meiji Yasuda Life Foundation of Health and Welfare (approval number: 2021–0001).

### 2.2 Experimental design of verbal n-back task

**Figure 1:**
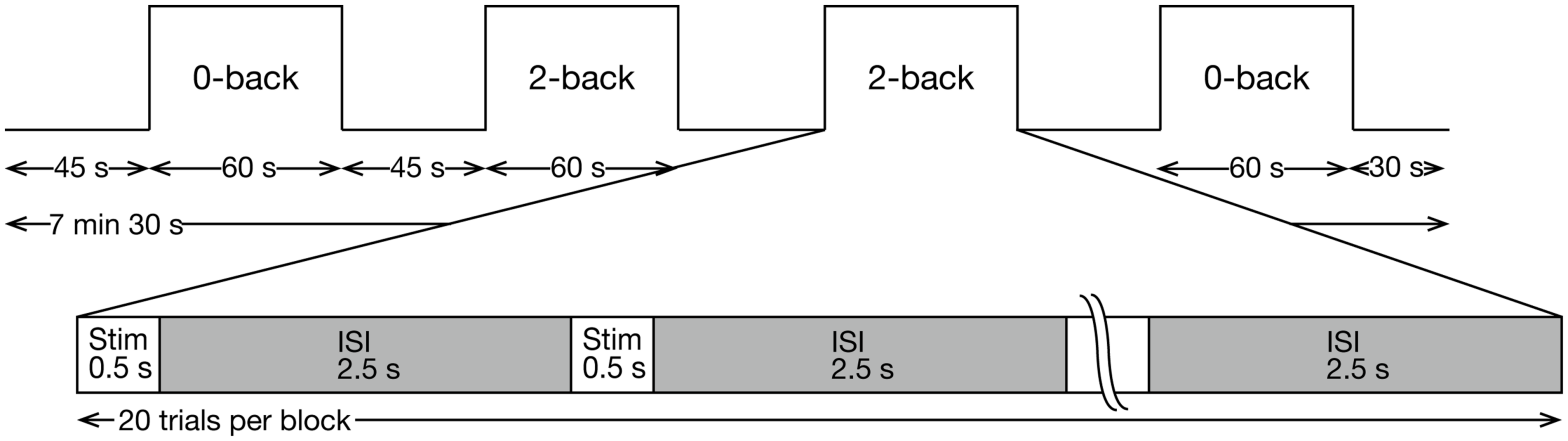
Experimental design of the *n*-back task. Each task block consisted of 20 trials (1 stimulus/trial) and was repeated twice in random order. Each stimulus was presented for 0.5 s, followed by an inter-stimulus interval (ISI) of 2.5 s. Each block lasted 60 s, preceded by a 45-s rest period. The total length of the *n*-back task was 7 min 30 s, including the 30-s rest period after the final task block.

Participants performed a verbal n-back task using hiragana characters (Figure 1), under two conditions: 0-back and 2-back. Each task condition consisted of two blocks, resulting in a total of four task blocks. Block order was randomized for each participant. Each block consisted of 20 trials (1 stimulus per trial), five of which were target trials. In the 0-back condition, participants were instructed to respond to a prespecified character as a target and to all other stimuli as non-targets. In the 2-back condition, participants were instructed to respond to stimuli whose character matched that presented two trials earlier as targets, and to all other stimuli as non-targets. Participants responded to target stimuli by pressing the ‘N’ key on a standard keyboard with their right index finger, and to non-target stimuli by pressing the ‘C’ key with their left index finger. Each task block lasted 60 s and was preceded by a 45-s rest period. The entire experiment lasted 7.5 min, including a 30-s rest period after the final block. Further details regarding the behavioral analysis are available in the Supplementary material.

### 2.3 fNIRS measurement

**Figure 2:**
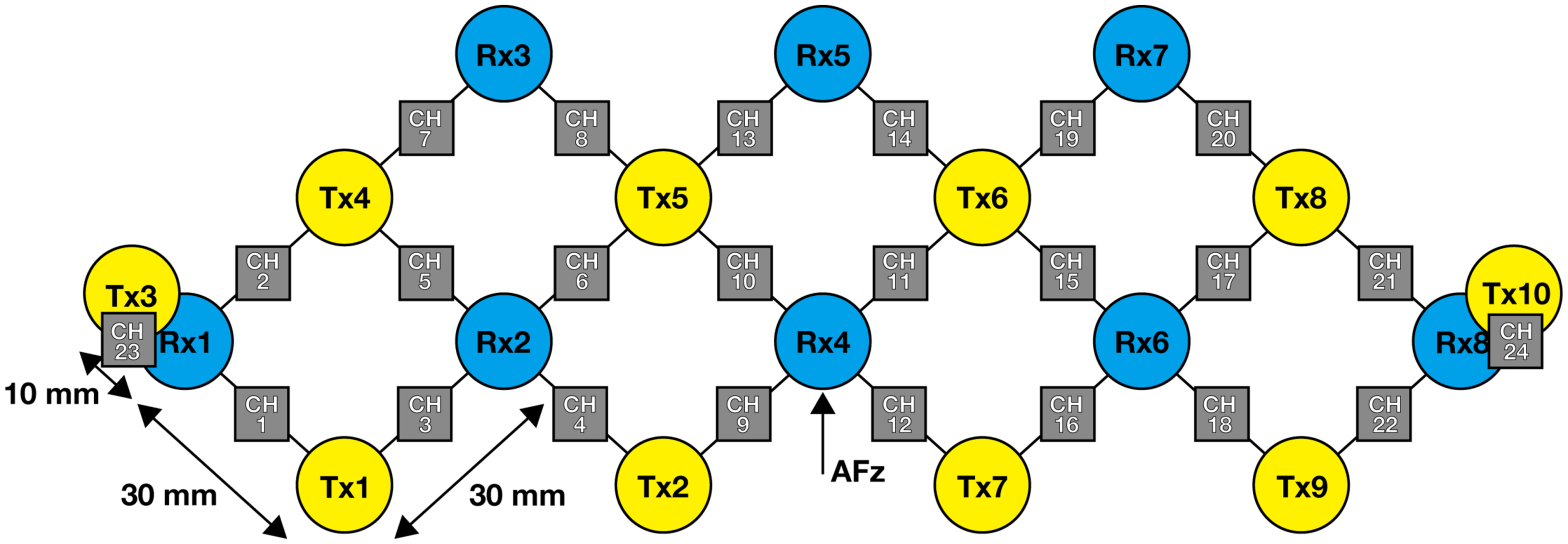
Optode configuration. Yellow circles indicate emitters, blue circles indicate detectors, and gray squares indicate channels (CHs). The numbers denote the identifiers of the emitters, detectors, and CHs. The emitter–detector separation was 30 mm for CHs 1–22 (long-separation CHs) and 10 mm for CHs 23 and 24 (short-separation CHs).

We used a Brite MKII (Artinis Medical Systems, The Netherlands) CW-NIRS device with 22 LS-CHs and two SS-CHs (seven LED emitters, λ_1|2_ = 760|850 nm, 10 photodiodes) sampled at 25 Hz. Data were converted into oxy- and deoxy-Hb signal changes using the modified Beer–Lambert law (Delpy et al., 1988) and were calculated into arbitrary units of mM × mm (Maki et al., 1995). The optode holder was positioned such that Rx4 was located at AFz, as illustrated in Figure 2. Each optode position was estimated using virtual registration (Tsuzuki et al., 2007) and was projected to MNI space using anchor-based registration (Tsuzuki et al., 2012). A detailed description of the algorithm used in the current virtual registration is presented in the Supplementary material. The LPBA40 atlas was used to anatomically label the locations of the estimated CHs (Shattuck et al., 2008).

### 2.4 fNIRS data preprocessing

**Figure 3:**
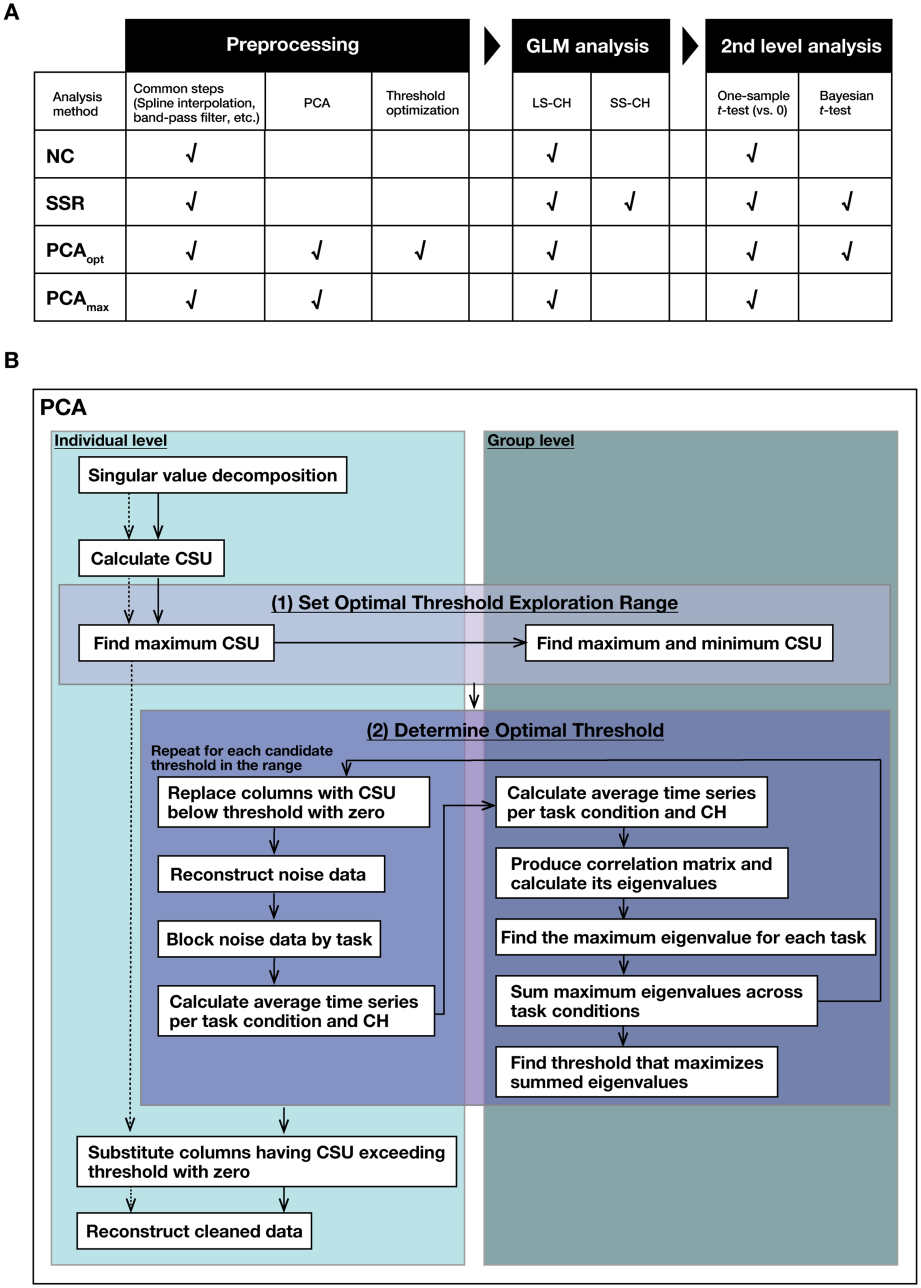
Flow of analysis. **(A)** Overview of the processing steps applied in each analysis method: no correction (NC), short-separation regression (SSR), principal component analysis (PCA) with threshold optimization (PCA_opt_), and PCA using the maximum value as the threshold (PCA_max_). The boxes with check marks indicate that the processing or analysis steps were included for the analysis method. **(B)** Flowchart of the PCA procedures (PCA_opt_ and PCA_max_) used in this study. Solid lines represent the processing flow for PCA_opt_, while dotted lines represent that for PCA_max_.

We used MatlabR2023b (The MathWorks, Inc., Natick, MA, USA) for fNIRS data analysis. Four analysis methods were employed: (1) NC, (2) SSR, (3) PCA_opt_, and (4) PCA_max_. The same preprocessing steps, described below, were applied prior to each method and were consistent across all analyses (Figure 3A). Data preprocessing to remove cardiac, respiratory, and motion artifacts was performed as follows. First, sudden spikes in the data were removed using spline interpolation (*p* = 0.01, W = 75, T = 0.15, amp = 0.5) (Scholkmann et al., 2010). Next, a band-pass filter (0.004—0.09 Hz) and linear correction using a first-degree polynomial were applied to the data. Subsequently, for each participant, CHs that showed no signal change for more than 10% of the entire time series were considered measurement deficits and were removed. CH 24, an SS-CH, was excluded from the analysis due to measurement deficits in more than 90% of the participants. Finally, wavelet-MDL (Jang et al., 2009) and temporal smoothing using the canonical hemodynamic response function (cHRF) (Friston et al., 2000) were applied to the data.

### 2.5 Extracerebral hemodynamics removal with PCA

For the PCA_opt_ and PCA_max_ analysis methods, extracerebral hemodynamics removal was performed using PCA following the preprocessing steps described above. In both methods, we first decomposed the fNIRS data using PCA and then calculated the CSU metric from each right-singular vector. PCA_opt_ thresholds for separating noise from signal were optimized as described in Section 2.6, below; for PCA_max_, the component with the maximum CSU for each participant was used as the threshold (Figure 3B). Finally, in both methods, components exceeding the respective thresholds were removed, and the signals were reconstructed. Here, we will describe the procedure common to both PCA_max_ and PCA_opt_, which includes fNIRS data decomposition, calculation of the CSU, and signal reconstruction after extracerebral hemodynamics removal.

The fNIRS data matrix for the k^th^ individual is denoted as H_k_, with dimensions *t* × *m*, where *t* is the number of time points and *m* is the number of CHs. We first performed a singular value.

Decomposition of the data as in the following Equation 1, where the data matrix is decomposed into three matrices: U_k_ (*t* × *t*), D_k_ (*t* × *m*), and V_k_ (*m* × *m*).


$$H_{k}=U_{k}D_{k}V_{k}^{T}$$ (1)

For each column of **V**_**k**_, which corresponds to each right-singular vector, the CSU, which is the metric indicating the extent of spatial uniformity among channels, was calculated. Each row of **V**_**k**_ corresponds to a right-singular vector. For each column, the CSU—a metric quantifying spatial uniformity among channels—was computed. Specifically, CSU is defined as the absolute value of the mean divided by the standard deviation of each column.


$$\;{\mathrm{CSU}}_{k}\;(j)=|\frac{\mu_{j}^{k}}{\sigma_{j}^{k}}|,\mu_{j}^{k}=\frac{1}{m}\sum_{i=1}^{m}v_{\mathrm{ij}}^{k},(j=1,2,\ldots m)$$ (2)

Let x be the threshold for separating signal from noise. The denoised matrix **V**_k_ removed is obtained by replacing the columns with CSU values greater than x (Equations 3–5). For PCA_opt_, the threshold x was determined as described in Section 2.6, below, whereas for PCA_max_, x was defined as the maximum CSU value for each individual.


$$\;V_{\text{removed},k}=[v_{k1}^{*},v_{k2}^{*},\ldots v_{\mathrm{km}}^{*}]$$ (3)

$$v_{\mathrm{kj}}^{*}=\{\begin{array}{c} v_{\mathrm{kj}}\;\;{\mathrm{CSU}}_{k}(j)<x\\ 0_{m}\;\;{\mathrm{CSU}}_{k}(j)\geq x \end{array}$$ (4)

$$H_{\text{removed},k}=U_{k}D_{k}V_{\text{removed},k}^{T}$$ (5)

### 2.6 Threshold optimization method

In the PCA_opt_ analysis, the threshold x was optimized specifically for this experimental design and for each participant based on the threshold optimization procedure described below. In essence, this threshold optimization method works in two steps: (1) setting the optimal threshold exploration range and (2) determining the optimal threshold x . The optimal threshold exploration range was determined according to the distribution of the maximum CSU values across all participants. Within the predefined optimal threshold exploration range, each candidate threshold value was applied to calculate noise components. For each channel in each candidate threshold value, these noise components were then averaged across participants. Finally, the optimal threshold x was defined from the candidate threshold value that yielded the highest inter-channel correlation of the identified noise components. Note that the optimal threshold in this study is defined as the value that removes components exhibiting high temporal commonality across all channels. This is based on the assumption that extracerebral hemodynamics are globally distributed across all CHs (Kohno et al., 2007; Sato et al., 2016). Therefore, components exhibiting high commonality across channels are interpreted as reflecting extracerebral hemodynamics, which are the target of the noise reduction. Here, we provide a detailed description of the procedures outlined above.

First, the optimal threshold exploration range was determined based on the distribution of CSU values across all data. We calculated the CSU of each column as described in Equation 2 in Section 2.5 and obtained the maximum CSU for each data as ***A*** = {max (CSU_1_), max (CSU_2_), …max (CSU*_k_*)}. Then, the minimum value [min(A)] and the maximum value [max (A)] of set A were determined as the boundaries for the optimal threshold exploration range. To determine the optimal threshold value, candidate thresholds were generated at 0.1 increments within the previously defined exploration range. A step size of 0.1 was selected to balance computational efficiency and optimization resolution, providing sufficient precision while avoiding unnecessary computational load. Next, we calculated the noise component for each candidate threshold. The new matrix, V_noise,*k*_, which includes noise components, can be calculated by subtracting the denoised matrix from the original matrix **V**_k_ as in Equation 6. The denoised data can be calculated with Equation 7 in which each candidate threshold value is substituted as threshold x . By recomposing the data using the new matrix, we obtained data with extracerebral hemodynamics H_noise,*k*_.


$$V_{\text{noise},k}=V_{k}-V_{\text{removed},k}$$ (6)

$$H_{\text{noise},k}=U_{k}D_{k}V_{\text{noise},k}^{T}$$ (7)

Following noise component calculation, averaged time series noise blocks were computed for each channel and task condition across all candidate thresholds. Each block used for averaging included the task period as well as 15 s each from the preceding and following rest periods. This duration was chosen to avoid temporal overlap between adjacent blocks. The baseline for each block was defined as the average signal during the 10 s preceding task onset. This baseline was subtracted from the time series of each block, and the resulting values were then averaged for each participant to generate individual averaged time series noise blocks for each task condition. After these calculations for each individual, we calculated the group-averaged time series noise blocks for each channel and task condition.

Next, we obtained the correlation matrix $M_{x}^{n-\text{back}}$ between each group-averaged time series noise block of channels for each task condition and each candidate threshold value. Then the eigenvalues of these correlation matrices were calculated, and we obtained the maximum eigenvalue, max( $\lambda_{x}^{n-\text{back}}$ ), for each task condition and each candidate threshold value. A large eigenvalue indicates that the main component of the group-averaged noise time series consists of signals that are highly correlated across channels. Finally, the sum of the maximum eigenvalues across all task conditions was computed. The optimum threshold was determined as the candidate threshold value yielding the largest sum of maximum eigenvalues across all candidate threshold values (Equations 8, 9).


$$\begin{array}{l} f(x)=\max(\lambda_{x}^{0-\text{back}})+\max(\lambda_{x}^{2-\text{back}}),\\ x\in [\min(A),\max(A)] \end{array}$$ (8)

$$f_{\mathrm{opt}}=\max(f(x))$$ (9)

### 2.7 GLM analysis

A GLM analysis was conducted following the common preprocessing procedure described in Section 2.4 and a PCA was conducted for the PCA_opt_ and PCA_max_ methods. We used the cHRF as the basis function, which was an HRF convolved with a boxcar function. We used the default values (τ_p_ = 6 s, τ_d_ = 10 s, A = 6) for the parameters of the HRF. For the analysis of deoxy-Hb, a negative cHRF was used to capture the inverse signal change associated with activation. In addition, the temporal and dispersion derivatives of the cHRF for each task condition were included as regressors to account for individual variability. A constant term was also added to the model. Furthermore, for the SSR analysis, data from one SS-CH was included as an additional regressor to account for extracerebral hemodynamics. From the GLM analysis, we obtained *β*-values, which represent the magnitude of activation, for each task condition. These β-values were used in the subsequent fNIRS statistical analysis.

### 2.8 fNIRS statistical analysis

Using the β-values derived from the GLM analysis, we examined differences in activation patterns across all analysis methods and compared activation levels specifically between the SSR and PCA_opt_ methods. Prior to the analyses, Z-scores of the β-values for each analysis method for each CH were calculated. Values with |Z| > 3.29 were regarded as outliers and were removed in a pairwise manner in the subsequent analyses. To clarify differences in the patterns of significant channels, one-sample t-tests (against zero) were conducted to identify significant activation for each task condition (0- and 2-back) with each analysis method (NC, SSR, PCA_opt_, and PCA_max_). We set the significance level at *α* = 0.05. Bonferroni correction was used for family-wise error correction. To demonstrate the absence of differences in activation detection between the two analysis methods, SSR and PCA_opt_, we conducted a series of Bayesian *t*-tests (Rouder et al., 2009). The results of the analysis were interpreted using the Bayes factor. In this analysis, a Bayes factor (BF_01_) greater than 1.0 indicated evidence in favor of the null hypothesis—providing evidence that the two analysis methods produce statistically indistinguishable results—whereas a BF_10_ less than 1.0 indicated evidence supporting the alternative hypothesis, suggesting that differences existed between the methods. The Bayes factor quantifies how much more likely the observed data are under one hypothesis compared to another. Unlike frequentist null hypothesis testing, which relies on a fixed significance level to reject a hypothesis, the Bayes factor provides a continuous measure of evidence for or against competing hypotheses. Thus, in a Bayesian analysis, there is no fixed threshold for rejecting or accepting a hypothesis (Hoijtink et al., 2019). However, in practice, a Bayes factor greater than 3.0 is interpreted as providing positive evidence for the favored hypothesis (Kass and Raftery, 1995). These Bayesian statistical analyses were conducted using R version 4.4.2 with the *bain* package (Gu et al., 2019).

## 3 Results

### 3.1 Optimized threshold value

The individual maximum CSU ranged from 0.5 to 4.5 for the oxy-Hb and 0.5 to 2.6 for the deoxy-Hb signals. The optimal threshold values in the current study were 1.9 for oxy-Hb and 1.2 for deoxy-Hb. Distribution of optimized threshold values across participants is shown in Supplementary Figure S6.

### 3.2 Activated channels for each analysis method

**Figure 4:**
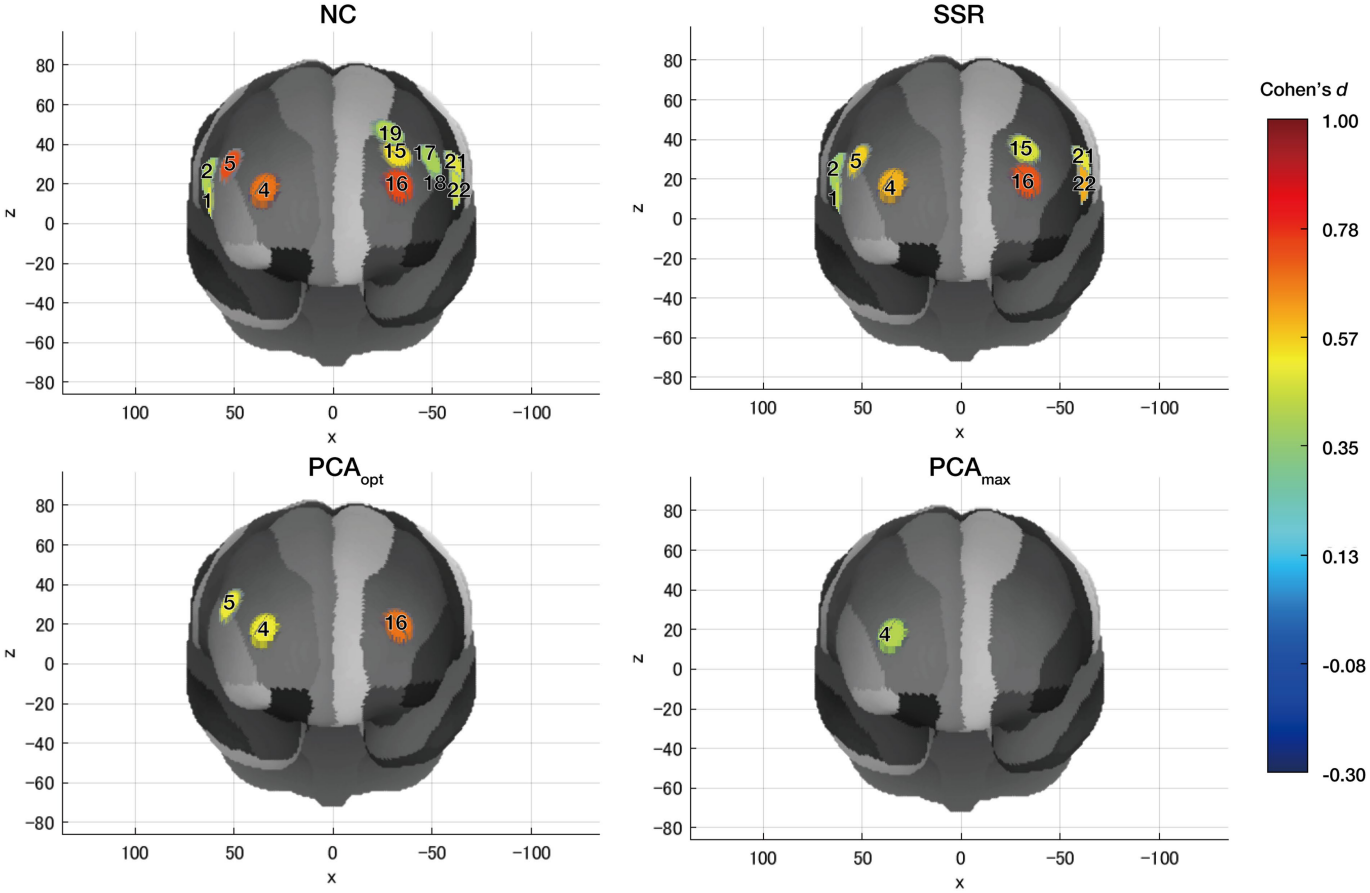
Significantly activated CHs for each analysis method for oxy-Hb in the 2-back condition: NC (top left), SSR (top right), PCA_opt_ (bottom left), and PCA_max_ (bottom right). Numbers indicate CH numbers. The color of each circle indicates the magnitude of activation, as shown in the color bar.

**Figure 5:**
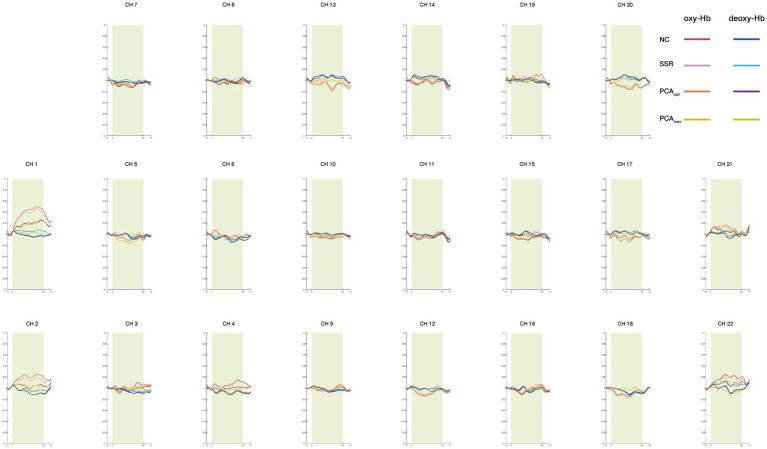
Averaged waveforms during the 0-back task for each analysis method for oxy-Hb and deoxy-Hb.

**Figure 6:**
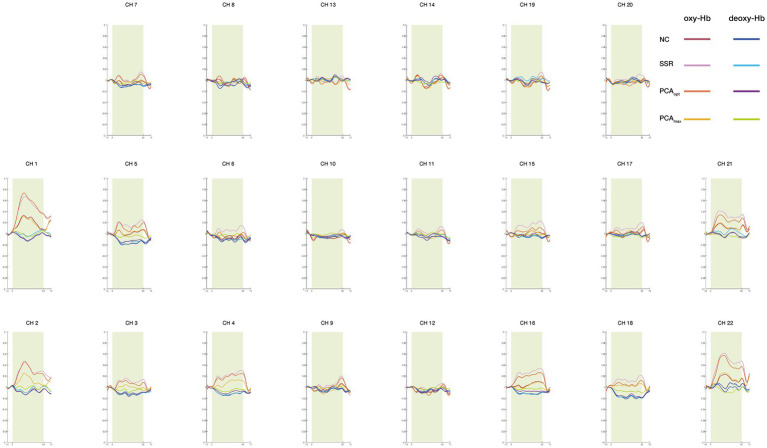
Averaged waveforms during the 2-back task for each analysis method for oxy-Hb and deoxy-Hb.

To examine differences in the distribution of significantly activated channels, one-sample *t*-tests (vs. 0) were performed for each task condition (0-back and 2-back) across all analysis methods (NC, SSR, PCA_opt_, and PCA_max_) to detect significant activations. Table 1 presents significantly activated channels for oxy-Hb. For the 2-back condition, differences between analysis methods are illustrated in Figure 4. The oxy-Hb and deoxy-Hb hemodynamic response waveforms for each channel are shown in Figures 5, 6. Deoxy-Hb results are presented in the Supplementary material.

**Table 1:** Results of one-sample *t*-tests (vs. 0) for channels significantly activated for oxy-Hb by at least one of the four analysis methods.

	CH	MNI coordinates			*SD* (mm)	Macro-anatomy		NC			SS			PCA_opt_			PCA_max_		
		*x*	*y*	*z*		LPBA40	(%)	*t*	*p*	*d*	*t*	*p*	*d*	*t*	*p*	*d*	*t*	*p*	*d*
0-back	20	−44.22	28.62	41.42	8.37	L middle frontal gyrus	100.00	−2.63	*n.s.*	−0.35	−2.05	*n.s.*	−0.27	−4.01	**	−0.53	−3.09	*n.s.*	−0.41
2-back	1	58.79	23.24	11.97	13.65	R inferior frontal gyrus	63.81	3.85	**	0.50	3.56	*	0.46	2.86	*n.s.*	0.37	0.56	*n.s.*	0.07
	2	59.45	17.03	23.46	11.13	R precentral gyrus	68.64	3.39	*	0.43	3.26	*	0.41	2.09	*n.s.*	0.27	1.22	*n.s.*	0.16
	4	32.92	61.89	15.89	5.98	R middle frontal gyrus	100.00	5.52	***	0.70	4.83	***	0.61	3.97	**	0.52	3.31	*	0.43
	5	49.19	37.66	27.69	7.46	R middle frontal gyrus	66.41	5.71	***	0.75	4.37	**	0.57	3.92	**	0.52	3.01	*n.s.*	0.39
	15	−30.02	51.37	33.04	6.08	L middle frontal gyrus	100.00	4.27	*	0.55	3.79	**	0.49	2.39	*n.s.*	0.32	1.34	*n.s.*	0.17
	16	−31.10	61.00	18.62	6.08	L middle frontal gyrus	100.00	6.05	***	0.76	5.80	***	0.74	5.32	***	0.70	2.11	*n.s.*	0.28
	17	−47.06	35.91	30.29	7.31	L middle frontal gyrus	100.00	3.26	*	0.41	2.96	*n.s.*	0.37	1.59	*n.s.*	0.21	1.82	*n.s.*	0.24
	19	−26.41	44.07	43.55	7.27	L middle frontal gyrus	73.04	3.37	*	0.43	2.25	*n.s.*	0.29	1.34	*n.s.*	0.18	0.79	*n.s.*	0.10
	21	−58.38	13.42	27.24	10.42	L precentral gyrus	50.96	3.92	**	0.50	4.03	**	0.51	2.52	*n.s.*	0.33	2.03	*n.s.*	0.27
	22	−59.17	17.78	17.70	10.38	L inferior frontal gyrus	79.35	3.76	**	0.47	4.88	***	0.62	2.59	*n.s.*	0.34	2.16	*n.s.*	0.28

L, left, R, right.

*p*-values are corrected for multiple comarisons using the Bonferroni correction. ***: *p* < 0.001, **: *p* < 0.01, *: *p* < 0.05, n.s.: non significant.

### 3.3 Bayesian analysis

Bayesian *t*-tests were conducted to examine the absence of differences between PCA_opt_ and SSR. Table 2 presents the Bayes factors for each CH and Supplementary Figure S7 presents their spatial distribution for oxy-Hb signals. In the 0-back task condition, all CHs except CH20 produced BF_01_ values greater than 3.0. In the 2-back task condition, approximately a quarter of all CHs produced BF_01_ values greater than 3.0. Twelve CHs had BF_01_ values between 1.0 and 3.0. Five CHs had BF_01_ values lower than 1.0 and their corresponding BF_10_ values were all below 3.0.

**Table 2:** Results of the Bayesian *t*-tests for the 0-back condition (top) and 2-back condition (bottom).

CH	1	2	3	4	5	6	7	8	9	10	11	12	13	14	15	16	17	18	19	20	21	22
0-back																						
BF_01_	7.51	7.21	4.51	7.42	5.37	5.87	7.61	3.59	6.04	7.68	6.94	4.3	3.65	7.48	6.24	3.09	6.9	5.78	5.46	0.92	6.94	6.25
BF_10_	0.13	0.14	0.22	0.13	0.19	0.17	0.13	0.28	0.17	0.13	0.14	0.23	0.27	0.13	0.16	0.32	0.14	0.17	0.18	1.09	0.14	0.16
2-back																						
BF_01_	7.48	3.67	2.59	1.39	2.85	0.72	1.49	5.62	1.34	2.24	1.05	0.61	1.41	5.68	1.63	1.99	0.47	0.68	0.76	0.04	1.8	2.56
BF_10_	0.13	0.27	0.39	0.72	0.35	1.4	0.67	0.18	0.75	0.45	0.95	1.64	0.71	0.18	0.61	0.5	2.13	1.47	1.32	25.2	0.56	0.39

## 4 Discussion

### 4.1 Overview of the current study

In the current study, we proposed a threshold optimization method that does not require SS-CHs for separating signal and noise components in order to remove the influence of extracerebral hemodynamics. This study focused on optimizing existing extracerebral hemodynamics removal methodologies, rather than proposing a new method, specifically by applying PCA with CSU as an index. In essence, the proposed threshold optimization method works as a two-step procedure following the decomposition of fNIRS signals using PCA: (1) defining the threshold exploration range and (2) selecting the optimal threshold. We evaluated whether applying PCA with the proposed threshold optimization method could effectively reduce the influence of extracerebral hemodynamics. We utilized data collected from participants performing an n-back task, which is a widely used cognitive task. Four analysis methods, NC, SSR, PCA_opt_, and PCA_max_, were each applied to the data. Our hypothesis was that if the proposed threshold optimization method successfully mitigated extracerebral hemodynamic influences, it should produce activation patterns comparable to those obtained using SSR. Therefore, we used the Bayesian *t*-test to confirm the absence of differences between the SSR and PCA_opt_ methods.

### 4.2 Differences in significant activation detection by analysis method

For the 0-back condition, significant deactivation in one CH was detected only for PCA_max_. For the 2-back condition, the NC method, as expected, yielded the highest number of significantly activated CHs, followed by SSR, PCA_opt_, and PCA_max_. In addition, the NC method produced the largest effect sizes. This is likely because it was the only analysis method that did not correct for extracerebral hemodynamics. The SSR method identified eight significantly activated CHs, which were included in the significantly activated CHs of the NC method, and produced smaller effect sizes than the NC method. These results indicate that there may be a risk of detecting false positives without correction for extracerebral hemodynamics. A lack of correction may also result in overestimation of the effect sizes relative to true cerebral activities. This further supports the necessity of adopting approaches to mitigate the influence of extracerebral hemodynamics, such as incorporating SS-CHs or applying proper statistical methods.

When comparing the significantly activated CHs between the PCA_max_ and PCA_opt_ methods, only one CH was detected with PCA_max_, whereas three were identified with PCA_opt_, including the one from PCA_max_. The activated CHs identified with both the PCA_max_ and PCA_opt_ methods were estimated to be located in the bilateral middle frontal gyrus (MFG). This indicates that cortical activation consistent with previous findings was elicited and accurately detected by both analysis methods. In contrast, PCA_opt_ identified additional activated CHs also located at the bilateral MFG, which were not detected with PCA_max_. This suggests that PCA_max_ may lead to underestimated cortical activation, producing false-negatives with regards to cortical activations. Consistent with this interpretation, the amplitude of the oxy-Hb signal obtained from the PCA_max_ method was much smaller than that from the PCA_opt_ method (Figure 6). Further, deactivation in the 0-back condition was only detected for this method, which indicates excess removal of oxy-Hb signals. This finding aligns with a study that revealed the possibility of overcorrecting for extracerebral hemodynamics when using currently available statistical methods instead of using SS-CHs (Klein et al., 2022).

It is possible that the conventional method of using the maximum value for each participant (PCA_max_) may have resulted in an underestimation of cortical activation, as it was based on the strict assumption that all individual data is contaminated by extracerebral hemodynamics. Using the PCA_opt_ method, data with a relatively small CSU within a dataset may end up with no components removed as extracerebral hemodynamics. Certainly, the reality is that there are no humans without extracerebral hemodynamics; therefore, it is unlikely that there is data in which no components need to be removed as extracerebral hemodynamics. Thus, in PCA_max_, an example analysis of the conventional usage of PCA without optimization, the component with the maximum CSU for each data was removed assuming it corresponded to extracerebral hemodynamics. However, the magnitude of this effect on fNIRS signals varies largely among individuals (Nasseri et al., 2018). Indeed, the distribution of the maximum CSU in this dataset showed considerable variation, ranging from a minimum of 0.5 to a maximum of 4.5. For the PCA_max_ analysis, the component with the maximum CSU was removed regardless of its value. This applied even when the maximum CSU was as low as 0.5 for an individual. However, a CSU value of 0.5 indicates low spatial uniformity and it is unlikely that the component contains extracerebral hemodynamics. Removal of these components may have resulted in overcorrections of fNIRS signals in the PCA_max_ method. Moreover, the optimum threshold values determined in this study were 1.9 for oxy-Hb and 1.2 for deoxy-Hb. These values were smaller than, equal to, or larger than those reported as threshold values in previous studies. This suggests that, even when using a fixed threshold value instead of the individual maximum, optimizing the threshold according to participants and experimental tasks may help prevent both overestimation and underestimation of cortical signals. Overall, when using statistical methods such as PCA instead of SS-CHs, threshold optimization based on the dataset is beneficial. This approach is preferable to using a fixed value or the individual maximum, as it reduces the risk of both false positive and false negative detection of cortical activations.

### 4.3 Bayesian analysis of SSR and PCA_opt_

We conducted Bayesian *t*-tests to assess the similarity between analysis methods by examining the absence of significant differences in activation magnitude for each CH detected by SSR and PCA_opt_. From the results of the Bayesian *t*-tests, we can conclude that PCA_opt_ is able to detect similar cortical activation as SSR in the 0-back condition. All CHs except one produced BF_01_ values greater than 3.0, providing positive support for the hypothesis that there are no differences in the detected cortical activations between SSR and PCA_opt_. In contrast, for the 2-back condition, Bayesian t-tests for most CHs did not provide positive support for the hypothesis. Consequently, we cannot conclude that PCA_opt_ can always detect activation similar to SSR. Given the low values of both BF₀₁ and BF₁₀ across most of the CHs in the 2-back condition, the results should be interpreted as providing no substantial evidence for either hypothesis. Differences between task conditions may reflect the expected effect size of cortical activation. Greater cortical activation is expected in the 2-back condition compared to the 0-back condition (Fishburn et al., 2014). Further, most of the measured CHs were located on the ROIs of the previous study (Hyodo et al., 2024). Thus, similar cortical activation may have occurred across many CHs, potentially leading to overcorrection of signals by PCA_opt_ compared to SSR. Conversely, because only one SS-CH was available, the current SSR method may have been insufficient to fully reflect extracerebral hemodynamics, which may have resulted in an undercorrection of signals compared to PCA_opt_ (see Section 4.4 for further discussion of this limitation). In summary of the Bayesian analysis, these findings suggest that PCA_opt_ may be able to detect similar activation as SSR. However, while their differences are not substantial, positive evidence supporting their equivalence is not consistently observed across task conditions. See also the Supplementary material for deoxy-Hb results.

### 4.4 Limitations and future prospectives of the study

There are five limitations to the current study. First, we assumed based on previous studies (Kohno et al., 2007; Sato et al., 2016) that extracerebral hemodynamics are spatially uniformly distributed. Although this assumption is used in multiple studies, other studies have adopted the opposite view that they are heterogeneously distributed (Gagnon et al., 2012; Wyser et al., 2020). The applicability of these assumptions and the associated assumed mechanisms underlying the effects of extracerebral hemodynamics remain controversial and are beyond the scope of our study. However, given that the area measured in this study was confined to the frontal, and partly to the temporal, regions, even if extracerebral hemodynamics are heterogeneously distributed, the influence of any such heterogeneity should be mitigated, especially compared to whole-head measurements. Future studies should consider whether assuming spatial homogeneity or heterogeneity of extracerebral hemodynamics will lead to optimal results. If assuming spatial heterogeneity may be more appropriate, the assumption we applied, removing components which exhibit spatial similarity across channels would no longer hold. In such cases, alternative approaches such as channel-specific threshold selection should be considered.

Second, due to measurement errors, we were able to use data from one of the two SS-CHs for analysis and as a regressor. Although there is another study that used only one SS-CH as a regressor (Gagnon et al., 2012), employing multiple SS-CHs is the more common approach (Gagnon et al., 2014; Noah et al., 2021; Sato et al., 2016). While availability of only a single SS-CH can become problematic if spatial heterogeneity of extracerebral hemodynamics is assumed, our assumption of homogeneous dispersion should mitigate it to a certain extent. Nevertheless, since we compared data from the SS-CH to validate our optimization method with PCA, there is a risk of overfitting to the SS-CH data from a single region. It is necessary to verify whether components derived from multiple SS-CHs yield results consistent with those derived from the current SSR results.

Third, although the current optimization method allows for dataset-specific threshold adjustment, there remains a risk of either overcorrection or undercorrection for extracerebral hemodynamics. One reason for overcorrection is the application of the method to small datasets with a limited number of CHs. This represents a common limitation of statistical approaches that do not utilize SS-CHs to reduce extracerebral hemodynamics. Specifically, if measured CHs covered only the ROI, cerebral activation is also likely to appear as a common component across CHs and within the dataset. As a result, true cerebral activity may be reduced, leading to overcorrection for extracerebral hemodynamics. As for underestimation, the current optimization method may retain data without any correction for extracerebral hemodynamics, potentially leading to an underestimation of their influence in some data. This was a practical approach to prevent overcorrection in our proposed method; however, it does not fully reflect real-world conditions, as there may be individuals who are only minimally affected by extracerebral hemodynamics. The present study demonstrates that, at the group level, this approach performs comparably to the current method of SSR. However, caution should be exercised when applying this method to individual-level analyses, such as those used in brain–computer interface applications or machine learning. Additionally, to minimize the risk of overcorrection when applying this method, optodes should be placed not only on ROIs but also in regions where task-related activity is not anticipated.

Fourth, we used a dataset obtained from an older adult population performing an n-back task. A previous study reported that the magnitude and spatial distribution of both cerebral activation and extracerebral hemodynamics varies with age (Schroeter et al., 2003). Furthermore, previous studies have shown that differences in experimental design, particularly those involving different ROIs, can lead to variations in the magnitude of extracerebral hemodynamics (Funane et al., 2014; Sato et al., 2016; Yücel et al., 2015). Therefore, the findings of our study may be limited to older adults and the n-back task. Further validation with other populations and with different experimental paradigms is needed in future research.

Fifth, our prosed optimization method was adapted to the use with PCA, in combination with CSU (Kohno et al., 2007) as an index. We decided to use this method since many studies have reported different threshold values when using CSU as an index (e.g., Virtanen et al., 2009). Nevertheless, several alternative methods for removing extracerebral hemodynamics have been proposed. Although the underlying principle of the proposed optimization, removing components with a value which exhibits the greatest uniformity across CHs, is not method-specific in theory, the current results may be specific to the extracerebral hemodynamics removal framework adopted in this study. Therefore, further validation and application using other approaches that assume spatial uniformity of extracerebral hemodynamics as well, such as the ICA-based method (Kohno et al., 2007), is necessary.

## 5 Conclusion

Taken together, optimizing the threshold for separating cerebral and extracerebral hemodynamics is an effective approach when applying PCA in the absence of SS-CH measurements. This approach allows a more appropriate correction compared to the conventional PCA approach, reducing the risk of overcorrection when PCA is used as a substitute for SS-CH data. While the proposed threshold optimizing method is adapted for use with PCA, in combination with CSU as an index, the underlying principle of optimizing the threshold is not method-specific and may be applicable to other ICA-based and PCA-based extracerebral hemodynamics removal methods, suggesting broader utilization. However, the generalizability of the results may be limited by the specific population and experimental design used in this study. Therefore, further research is needed to determine whether this method is applicable to other populations and experimental paradigms. Such exploration will enable appropriate correction with minimal risk of over- or undercorrection for fNIRS data without SS-CH measurements, thereby helping to prevent false-positive or false-negative detection of cortical activation.

## Data Availability

The raw data supporting the conclusions of this article will be made available by the authors, without undue reservation.
