# Supplementary material for: Threshold optimization in separating cortical and extracerebral hemodynamics using principal component analysis
Source: Front Hum Neurosci. 2026 Apr 22;20:1778201. doi: 10.3389/fnhum.2026.1778201 (PMC13143948; doi:10.3389/fnhum.2026.1778201)
Supplement: Supplementary file 1 [file Data_Sheet_1.pdf]

# *Supplementary Material*

## 1. Virtual registration

### 1.1 What is virtual registration?

To ensure comparability of the fNIRS data with results from other studies, it is necessary to project the data onto MNI space. This is easy if the MRI data of the participants is available. However, obtaining MRI data during an fNIRS experiment is often challenging due to limited time and financial resources. Instead, it is common to measure the optode positions on the scalp using a 3D digitizer or similar device and to estimate the channel locations in MNI space via probabilistic registration based on those measurements (Tsuzuki et al., 2012). However, a drawback of this method is that it requires physical measurement of the optode positions.

To address this limitation, virtual registration was introduced to enable registration of fNIRS data without a 3D digitizer (Tsuzuki et al., 2007). This method estimates the positions of optodes on the scalp through simulation, taking into account the deformation of each optode holder. Algorithms have been developed for Shimadzu's fixed optode holder and Hitachi's elastic optode holder.
Since this method was proposed in 2007, headcap-type optode holders—differing in material and shape from both fixed and elastic optode holders—have emerged as another conventional optode holder type. It is not difficult to imagine that the deformation of the headcap-type optode holder differs from that of previously developed optode holders due to the distinct material properties. Therefore, our objective here was to develop a virtual registration algorithm to estimate the optode positions when a headcap-type optode holder was worn, specifically with optodes placed on the frontal and part of the temporal regions of the head.

### 1.2 Placements of optodes on headcap-type optode holders

Fixed and elastic optode holders are typically designed with a predetermined optode arrangement, such as 3×5. In contrast, headcap-type holders support various optode configurations using a single type of holder. An Artinis headcap optode holder was used in the present study, with 17 optodes placed on the frontal and part of the temporal regions. For a detailed image of the optode configuration, see Fig. 2 in the main manuscript. AFz was used as the reference point for optode placement in the present study.

### 1.3 Virtual registration algorithm for the Artinis headcap type optode holder

When an Artinis headcap optode holder is worn, it deforms to conform to various head sizes and shapes: it deforms by stretching along a direction that runs parallel to the diagonal line formed by the optodes extending from the reference point, and it stretches along a nearly horizontal line through the reference point, keeping a constant angle relative to the midline. Four guidelines were established for the present virtual registration algorithm based on these deformation patterns (Fig. S1). The third guideline, illustrated in Fig. S1C, was used only to determine the position of the optode on the midline, while an optode-to-optode length of 30 mm was prioritized in determining the coordinates of the other optodes.

Our virtual registration algorithm was developed based on these four guidelines. We will now describe the algorithm (Fig. S2) in detail. First, the reference optode (Rx4) is positioned at AFz. The midline, serving as the reference arc, is then defined by connecting AFz and Cz (Fig. S2A). Next, the coordinate of the other optode on the midline (Rx5) is determined. The distance between the two optodes is calculated using the cosines of the fixed angles $q_T$ and $q_B$ and the position of the Rx5 optode is fixed along the reference arc (Fig. S2B). Next, a second, nearly horizontal, reference arc is drawn by

rotating the original reference arc according to the fixed angles $q_R$ and $q_B$ (Fig. S2C). The subsequent procedures (Fig. S2D-J) are then repeated until coordinates for all optodes are determined. The coordinates of the optodes are determined in sequence, proceeding from the center (reference point Rx4) toward the periphery; please see Fig. S2 for details. When placing optodes, if the next optode to be positioned must be 30 mm away from two already fixed optodes, its coordinates are determined as the point located 30 mm from both (e.g., Fig. S2D). However, if the next optode to be positioned must be 30 mm away from only one already fixed optode, its coordinates are determined accordingly. In that case, the optode is positioned such that it lies 30 mm from the fixed optode and aligns with one of the reference arcs (e.g., Fig. S2E).

### 1.4 Validating the virtual registration algorithm for the Artinis headcap-type optode holder

To evaluate the validity of the current algorithm, we compared the estimated optode positions with actual data. Similar to a previous study (Tsuzuki et al., 2007), we generated synthetic heads, placed the optodes using the algorithm, and projected them onto MNI space. This procedure was repeated 1000 times. Then the average coordinates and standard deviations (SDs) were calculated. For the actual data, optode positions on five participants who were not involved in the fNIRS measurements were measured using a 3D digitizer. The data were projected to MNI space, and the mean coordinates and SDs in MNI space were calculated. The root mean square error (RMSE) of the optode positions was calculated to

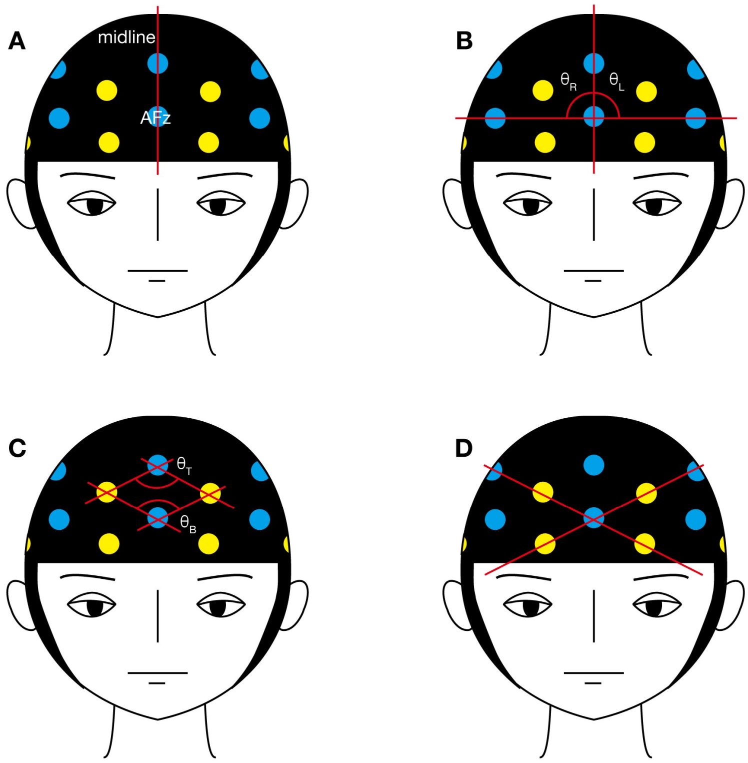


**Fig. S1** Four guidelines used for the current Artinis holder and optode configuration. (A) Rx4 is positioned at AFz, and both Rx4 and Rx5 are aligned along the midline. (B) The lower receiver optode arrays intersect the midline at angles $q_R$ and $q_L$, respectively. (C) The interior angles formed at Rx4 and Rx5 are fixed at $q_B$ and $q_T$, respectively. (D) The optodes arranged diagonally from Rx4 form an arc.

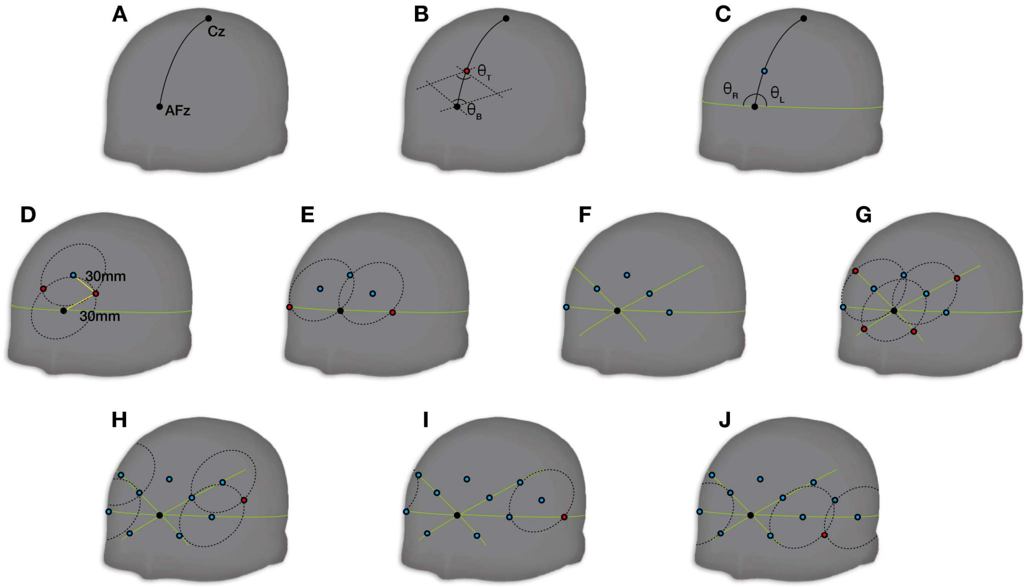


**Fig. S2** Virtual deformation algorithm for the current Artinis holder. (A) The reference optode (black dot), Rx4 in this case, is placed on AFz. A reference arc is then drawn connecting AFz and Cz. (B) The distance between Rx4 and Rx5 is determined by calculating the cosines of the fixed angles $q_T$ and $q_B$. The position of Rx5 is then fixed at that distance along the reference arc. (C) The reference arc is rotated based on the fixed angles $q_R$ and $q_B$, and an approximately horizontal arc is drawn. (D) Two points located 30 mm from the two previously determined optode positions (Rx4 and Rx5) are obtained and optodes are placed at these points. (E) Two optodes are placed at points 30 mm from the optode positions determined in (D) and located on the approximately horizontal arc defined in (C). (F) Two diagonal arcs that pass through the optodes determined in (D) and the reference optode Rx4 are drawn. (G) Optodes are placed at points on the arcs determined in (F), located 30 mm from the positions of either the reference optode or the optodes determined in (D). (G) Three optodes are placed at points 30 mm from the positions of either the reference optode or the optodes determined in (D & E) and located on the diagonal arc defined in (F). (H) Two points, each located 30 mm from the optode positions determined in (E & G), are obtained, and optodes are placed at these points (left side is shown). (I) Two optodes are placed at points 30 mm from the optode positions determined in (H) and located on the approximately horizontal arc defined in (C) (left side is shown). (J) Two points, each located 30 mm from the optode positions determined in (E & I), are obtained, and optodes are placed at these points (left side is shown).

compare the outcomes from virtual and actual registration. Prior to this calculation, the virtual coordinates were transformed so that the reference point matched that of the actual registration.

### 1.5 Results of validation

Optode positions on the head obtained through actual and virtual registration were compared to evaluate the accuracy of the proposed virtual registration algorithm. As illustrated in Fig. S3 (left), the positions of the virtually registered optodes generally matched those obtained from the actual measurements. SDs ranged from 7.08 to 9.98 mm for actual registration and from 5.68 to 17.3 mm for virtual registration. The RMSE between the actual and virtual optode positions was 4.00 mm. Similarly, optode positions on the cortical surface obtained through actual and virtual registration were compared. As with the results on the head surface, the positions of the virtually registered optodes on the cortical surface, as illustrated in Fig. S3 (right), generally matched those obtained from the actual measurements. SDs ranged from 5.77 to 9.17 mm for actual registration and from 5.09 to 14.2 mm for virtual registration. The RMSE between the actual and virtual optode positions was 4.15 mm.

## 1.6 Discussion

These results indicate that the proposed virtual registration algorithm was able to estimate optode positions comparable to those obtained through actual registration. While the range in SDs for virtual registration was larger than that for actual registration, it was comparable to SD ranges reported for virtual registration methods using fixed or elastic optode holders in previous studies. Thus, it can be concluded that the proposed virtual registration algorithm allows the estimation of optode locations using virtual heads of various sizes. For the estimated optode locations, those obtained by virtual registration fell within the standard deviation range of actual registration. This suggests that the proposed algorithm can estimate optode locations with practical accuracy.

However, the differences between actual and virtual registrations were larger than those reported for fixed or elastic holders in the previous study[30]. This may be due to the tendency for standard deviations to increase for optodes farther from the reference optode located at AFz in both algorithms, resulting in larger differences for those distant optodes. This tendency has been observed only for the current headcap-type optode holders and not for fixed or elastic optode holders. Thus, the larger difference between the registration methods may reflect the deformation characteristics specific to the Artinis headcap-type optode holders.

Three limitations inherent to the methodology should be noted. First, the fixed angles used in the proposed algorithm were determined using the same dataset that was later employed to validate the results of the virtual registration. This is because only five actual sets of registration data were available. Thus, further verification of the algorithm with additional actual registration data is required. Second, the results may be limited to the current optode configuration using AFz as the reference point. The region of interest in this study was the prefrontal cortex, including parts of the temporal regions. Other areas, such as the parietal and occipital cortices, were beyond the scope of this study. Thus, we did not verify the algorithm for those areas. Further verification and application of the algorithm for other brain regions or whole-head measurements are needed. Finally, the algorithm may be limited to head-cap type optode holders from Artinis. Since the fNIRS system used in the current study was the Artinis MK-2, we developed a virtual registration algorithm specifically for the Artinis head-cap type optode holder. However, the materials and characteristics of optode holders may vary between manufacturers, even for the same type of headcap-type optode holder. Thus, further verification of the algorithm is needed for other headcap-type optode holders from different manufacturers.

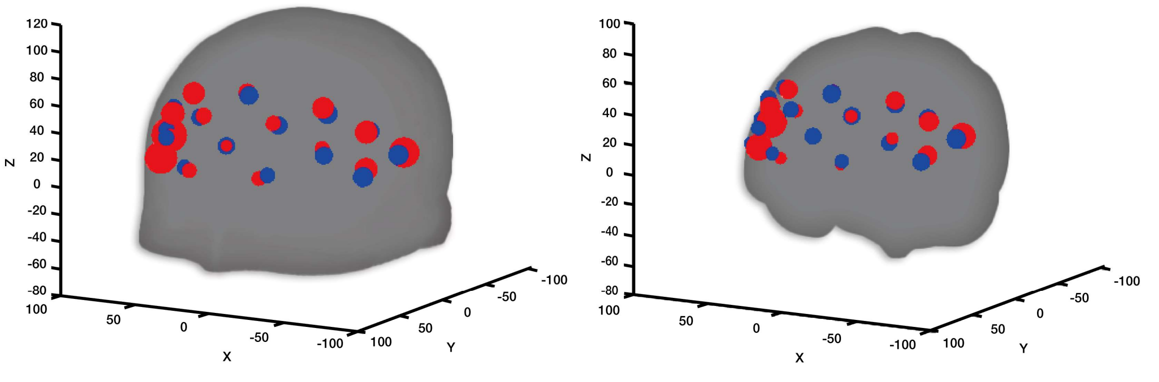


**Fig. S3** Estimated optode locations and SDs for actual and virtual registration on the head (left) and cortical (right) surfaces. Estimated locations from actual and virtual registrations are shown as blue and red circles, respectively, with circle size indicating the SD.

## 2. Behavioral analysis

### 2.1 Methods

To confirm that participants were appropriately engaged in the n-back task and that the two task conditions functioned as intended, we analyzed the behavioral data. If participants were appropriately engaged and the task conditions functioned as intended, reaction times (RTs) were expected to be longer and accuracy (ACC) lower in the 2-back task condition compared to the 0-back task condition. Trial-by-trial data on RTs and correct/incorrect responses were collected for both conditions.

For RTs, the mean RT for correctly answered target trials was calculated for each task condition. RTs that were more than two standard deviations from the mean were considered outliers and excluded from the analysis. Paired $t$-tests were conducted to examine differences in RTs and ACC between the two task conditions.

### 2.2 Results and discussion

RTs for the 2-back task were significantly longer than those for the 0-back task, and ACC was significantly lower in the 2-back task compared to the 0-back task. These results indicate that participants were appropriately engaged in the experimental task and that the task conditions functioned as intended.

## 3. Analysis of deoxy-Hb

### 3.1 Significant activation for each task condition

To examine differences in the distribution of significantly activated channels, one-sample $t$-tests (vs. 0) were performed for each task condition (0-back and 2-back) across all analysis methods (NC, SSR, $PCA_{opt}$, and $PCA_{max}$) for the deoxy-Hb data to detect significant activations. Table S1 presents the significantly activated channels (CHs) based one-sample $t$-tests for the 0-back and 2-back conditions for deoxy-Hb. For the 2-back condition, differences between analysis methods are illustrated in Fig. S5.

### 3.2 Bayesian analysis

To demonstrate the absence of differences in detected activation between $PCA_{opt}$ and SSR, we conducted Bayesian $t$-tests. Table S2 presents the Bayes factors for each CH for deoxy-Hb and Supplementary Figure S8 presents their spatial distribution for deoxy-Hb signals. In the 0-back condition, more than half of the CHs showed $BF_{01}$ values greater than 3.0. Five CHs showed $BF_{01}$ values between 1.0 and 3.0. Three CHs showed $BF_{01}$ values lower than 1.0. In the 2-back condition, more than half of the CHs showed $BF_{01}$ values greater than 3.0 Four CHs showed $BF_{01}$ values between 1.0 and 3.0. Four CHs showed $BF_{01}$ values lower than 1.0.

### 3.3 Discussion

In summary, the deoxy-Hb results generally support the findings derived from oxy-Hb. Consistent with the oxy-Hb results, the number of significantly activated CHs was the highest for NC, followed by SSR, $PCA_{opt}$, and $PCA_{max}$. The effect sizes across CHs generally followed this trend, regardless of task

condition. The findings that the NC method yielded the highest number of significant CHs and the largest effect sizes are consistent with findings from oxy-Hb, supporting the risk of detecting false positives without correction for extracerebral hemodynamics.

When comparing significantly activated CHs between $PCA_{opt}$ and $PCA_{max}$, the results in the 0-back condition were similar between the two methods. However, in the 2-back condition, $PCA_{opt}$ revealed one additional significant CH with greater activation compared to $PCA_{max}$. This further supports the findings from oxy-Hb, suggesting that PCA without threshold optimization may lead to false-negative detection of cortical activation.

Based on the results of the Bayesian *t*-tests, it can be concluded that $PCA_{opt}$ appears to be generally capable of detecting cortical activation patterns similar to those identified by SSR in both task conditions. This is supported by the finding that more than half of the CHs provided positive evidence for the null hypothesis indicating no difference between SSR and $PCA_{opt.}$ Still, some CHs provided no substantial evidence for either hypothesis and one CH provided positive evidence supporting the alternative hypothesis for each task condition. Similar to the oxy-Hb results, these inconsistencies may be attributable to methodological limitations of the present study, including the relatively limited spatial coverage of the measurement area in relation to the expected activation region and the availability of only one SS-CH.

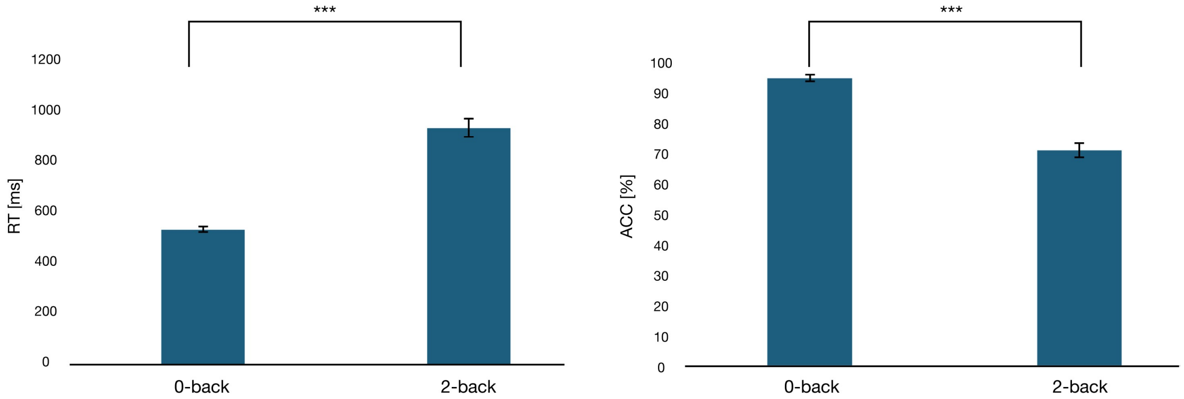


**Fig. S4** Differences in RT (left) and ACC (right) across task conditions. ***: $p < .001$. Error bars represent standard errors.

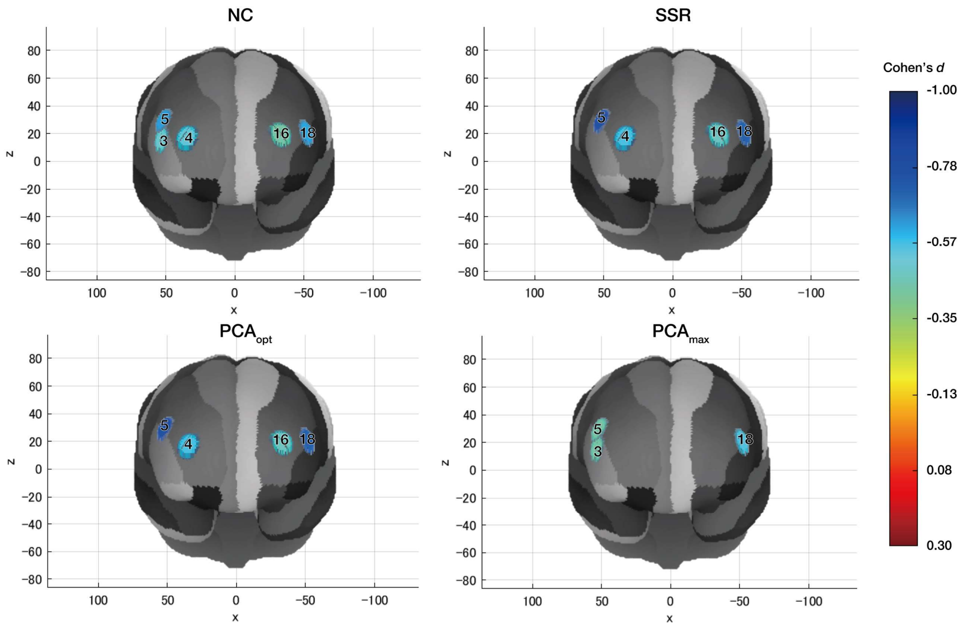


**Fig. S5** Significantly activated CHs for each analysis method for deoxy-Hb in the 2-back condition: NC (top left), SSR (top right), $PCA_{opt}$ (bottom left), and $PCA_{max}$ (bottom right). Numbers indicate CH numbers. The color of each circle indicates the magnitude of activation, as shown in the color bar.

# 4. Supplementary figures and tables

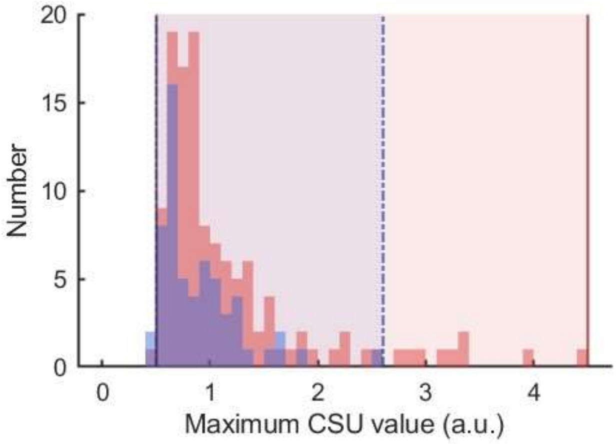


**Fig. S6** Histogram of maximum CSU values. Red bars indicate oxy-Hb, and blue bars indicate deoxy-Hb. The red solid lines and shaded area represent the exploration range for oxy-Hb, whereas the blue dashed lines and shaded area represent the exploration range for deoxy-Hb.

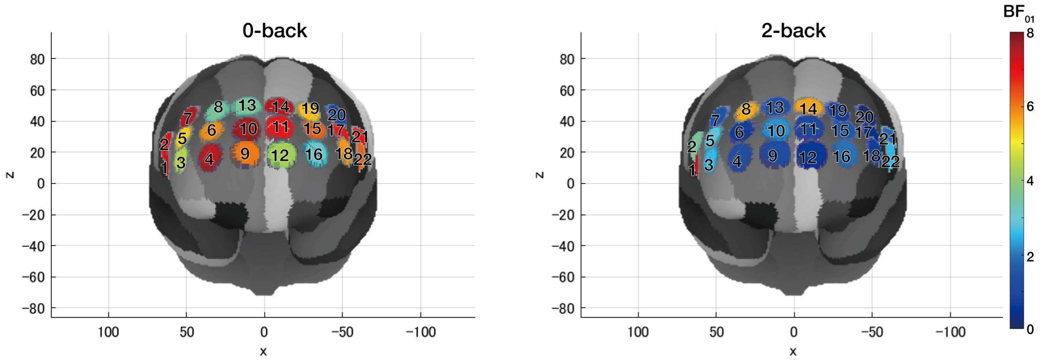


**Fig. S7** Spatial distribution of $BF_{01}$ values for oxy-Hb. The color of each circle indicates the magnitude of BF01 values, as shown in the color bar.

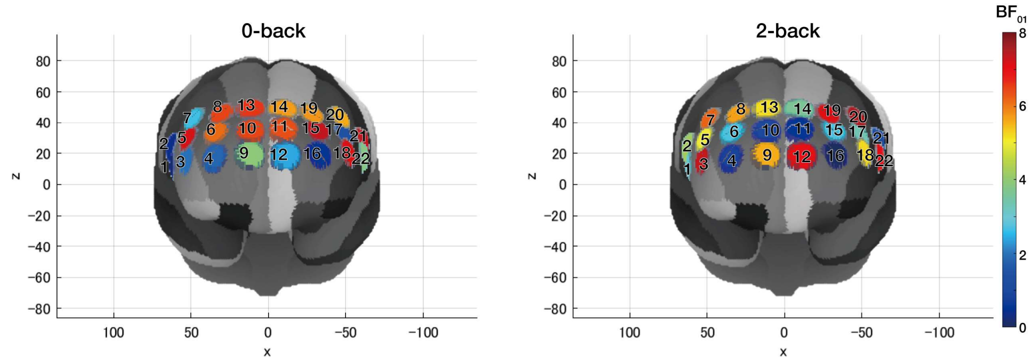


**Fig. S8** Spatial distribution of $BF_{01}$ values for deoxy-Hb. The color of each circle indicates the magnitude of BF01 values, as shown in the color bar.

**Table S1** Results of one-sample t-tests (vs. 0) for channels significantly activated for deoxy-Hb by at least one of the four analysis methods.

| | CH | MNI coordinates | | | *SD* (mm) | LPBA40 | (%) | NC | | | SS | | | PCAopt | | | PCAmax | | |
|---|---|---|---|---|---|---|---|---|---|---|---|---|---|---|---|---|---|---|---|
| | | *x* | *y* | *z* | | | | *t* | | *d* | *t* | | *d* | *t* | | *d* | *t* | | *d* |
| **0-back** | 3 | 50.08 | 43.43 | 14.81 | 9.25 | R inferior frontal gyrus | 85.71 | 3.37 | * | 0.43 | 3.11 | *n.s.* | 0.39 | 2.98 | *n.s.* | 0.39 | 1.35 | *n.s.* | 0.18 |
| | 4 | 32.92 | 61.89 | 15.89 | 5.98 | R middle frontal gyrus | 100.00 | 4.20 | * | 0.53 | 3.92 | ** | 0.49 | 3.87 | ** | 0.50 | 3.86 | ** | 0.50 |
| | 6 | 32.29 | 53.29 | 31.77 | 6.42 | R middle frontal gyrus | 100.00 | 4.14 | ** | 0.54 | 4.52 | *** | 0.60 | 3.86 | ** | 0.51 | 3.77 | ** | 0.50 |
| | 18 | -49.26 | 40.06 | 19.83 | 7.55 | L middle frontal gyrus | 61.44 | 3.25 | * | 0.43 | 3.11 | *n.s.* | 0.40 | 2.94 | *n.s.* | 0.39 | 1.35 | *n.s.* | 0.18 |
| **2-back** | 3 | 50.08 | 43.43 | 14.81 | 9.25 | R inferior frontal gyrus | 85.71 | 3.97 | ** | 0.50 | 3.02 | *n.s.* | 0.38 | 3.37 | ** | 0.44 | 3.25 | * | 0.43 |
| | 4 | 32.92 | 61.89 | 15.89 | 5.98 | R middle frontal gyrus | 100.00 | 4.19 | *** | 0.53 | 4.43 | *** | 0.56 | 3.57 | *** | 0.46 | 2.70 | *n.s.* | 0.35 |
| | 5 | 49.19 | 37.66 | 27.69 | 7.46 | R middle frontal gyrus | 66.41 | 4.71 | *** | 0.62 | 5.79 | *** | 0.76 | 4.06 | ** | 0.54 | 3.24 | * | 0.43 |
| | 16 | -31.10 | 61.00 | 18.62 | 6.08 | L middle frontal gyrus | 100.00 | 3.41 | * | 0.43 | 3.79 | ** | 0.48 | 1.98 | *n.s.* | 0.26 | 1.38 | *n.s.* | 0.18 |
| | 18 | -49.26 | 40.06 | 19.83 | 7.55 | L middle frontal gyrus | 61.44 | 4.63 | *** | 0.61 | 5.38 | *** | 0.71 | 4.01 | ** | 0.53 | 4.20 | *** | 0.55 |

*Notes.* P-values are corrected for multiple comparisons using the Bonferroni correction. Cortical region abbreviations: middle frontal gyrus (MFG), inferior frontal gyrus (IFG), superior frontal gyrus (SFG), precentral gyrus (PreCG), left (L), right (R).

**Table S2** Results of the Bayesian t-tests for the 0-back condition (top) and 2-back condition (bottom).

0-back

| CH | 1 | 2 | 3 | 4 | 5 | 6 | 7 | 8 | 9 | 10 | 11 | 12 | 13 | 14 | 15 | 16 | 17 | 18 | 19 | 20 | 21 | 22 |
|---|---|---|---|---|---|---|---|---|---|---|---|---|---|---|---|---|---|---|---|---|---|---|
| $BF_{01}$ | 0.19 | 0.38 | 1.98 | 1.84 | 7.22 | 6.16 | 2.67 | 6.52 | 3.95 | 6.5 | 6.4 | 2.41 | 6.65 | 5.76 | 7.35 | 0.56 | 1.47 | 7.19 | 5.8 | 5.63 | 7.41 | 3.83 |
| $BF_{10}$ | 5.26 | 2.64 | 0.5 | 0.54 | 0.14 | 0.16 | 0.38 | 0.15 | 0.25 | 0.15 | 0.16 | 0.41 | 0.15 | 0.17 | 0.14 | 1.78 | 0.68 | 0.14 | 0.17 | 0.18 | 0.13 | 0.26 |

2-back

| CH | 1 | 2 | 3 | 4 | 5 | 6 | 7 | 8 | 9 | 10 | 11 | 12 | 13 | 14 | 15 | 16 | 17 | 18 | 19 | 20 | 21 | 22 |
|---|---|---|---|---|---|---|---|---|---|---|---|---|---|---|---|---|---|---|---|---|---|---|
| $BF_{01}$ | 2.93 | 4.27 | 7.38 | 0.83 | 5 | 2.73 | 6.22 | 5.71 | 5.6 | 0.95 | 0.56 | 7.04 | 5.2 | 3.66 | 2.75 | 0.04 | 3.35 | 5.24 | 7.14 | 7.54 | 1.35 | 7.06 |
| $BF_{10}$ | 0.34 | 0.23 | 0.14 | 1.2 | 0.2 | 0.37 | 0.16 | 0.18 | 0.18 | 1.05 | 1.77 | 0.14 | 0.19 | 0.27 | 0.36 | 27.3 | 0.3 | 0.19 | 0.14 | 0.13 | 0.74 | 0.14 |

**Table S3** Results of the sensitivity analysis. The first and second rows correspond to oxy-Hb, and the third and fourth rows correspond to deoxy-Hb. Odd-numbered rows represent the 0-back condition, and even-numbered rows represent the 2-back condition.

0-back

| CH | 1 | 2 | 3 | 4 | 5 | 6 | 7 | 8 | 9 | 10 | 11 | 12 | 13 | 14 | 15 | 16 | 17 | 18 | 19 | 20 | 21 | 22 |
|---|---|---|---|---|---|---|---|---|---|---|---|---|---|---|---|---|---|---|---|---|---|---|
| bg | 7.51 | 7.21 | 4.51 | 7.42 | 5.37 | 5.87 | 7.61 | 3.59 | 6.04 | 7.68 | 6.94 | 4.3 | 3.65 | 7.48 | 6.24 | 3.09 | 6.9 | 5.78 | 5.46 | 0.92 | 6.94 | 6.25 |
| bg2 | 5.31 | 5.1 | 3.19 | 5.24 | 3.8 | 4.15 | 5.38 | 2.54 | 4.27 | 5.43 | 4.91 | 3.04 | 2.58 | 5.29 | 4.41 | 2.18 | 4.88 | 4.09 | 3.86 | 0.65 | 4.9 | 4.42 |
| bg3 | 4.34 | 4.16 | 2.6 | 4.28 | 3.1 | 3.39 | 4.4 | 2.07 | 3.49 | 4.43 | 4.01 | 2.48 | 2.11 | 4.32 | 3.6 | 1.78 | 3.98 | 3.34 | 3.15 | 0.53 | 4 | 3.61 |

2-back

| CH | 1 | 2 | 3 | 4 | 5 | 6 | 7 | 8 | 9 | 10 | 11 | 12 | 13 | 14 | 15 | 16 | 17 | 18 | 19 | 20 | 21 | 22 |
|---|---|---|---|---|---|---|---|---|---|---|---|---|---|---|---|---|---|---|---|---|---|---|
| bg | 7.48 | 3.67 | 2.59 | 1.39 | 2.85 | 0.72 | 1.49 | 5.62 | 1.34 | 2.24 | 1.05 | 0.61 | 1.41 | 5.68 | 1.63 | 1.99 | 0.47 | 0.68 | 0.76 | 0.04 | 1.8 | 2.56 |
| bg2 | 5.29 | 2.6 | 1.83 | 0.98 | 2.01 | 0.51 | 1.05 | 3.98 | 0.95 | 1.58 | 0.74 | 0.43 | 1 | 4.02 | 1.15 | 1.41 | 0.33 | 0.48 | 0.53 | 0.03 | 1.27 | 1.81 |
| bg3 | 4.32 | 2.12 | 1.5 | 0.8 | 1.65 | 0.41 | 0.86 | 3.25 | 0.77 | 1.29 | 0.61 | 0.35 | 0.82 | 3.28 | 0.94 | 1.15 | 0.27 | 0.39 | 0.44 | 0.02 | 1.04 | 1.48 |

0-back

| CH | 1 | 2 | 3 | 4 | 5 | 6 | 7 | 8 | 9 | 10 | 11 | 12 | 13 | 14 | 15 | 16 | 17 | 18 | 19 | 20 | 21 | 22 |
|---|---|---|---|---|---|---|---|---|---|---|---|---|---|---|---|---|---|---|---|---|---|---|
| bg | 0.19 | 0.38 | 1.98 | 1.84 | 7.22 | 6.16 | 2.67 | 6.52 | 3.95 | 6.5 | 6.4 | 2.41 | 6.65 | 5.76 | 7.35 | 0.56 | 1.47 | 7.19 | 5.8 | 5.63 | 7.41 | 3.83 |
| bg2 | 0.13 | 0.27 | 1.4 | 1.3 | 5.1 | 4.35 | 1.89 | 4.61 | 2.8 | 4.6 | 4.53 | 1.71 | 4.7 | 4.07 | 5.2 | 0.4 | 1.04 | 5.08 | 4.1 | 3.98 | 5.24 | 2.71 |
| bg3 | 0.11 | 0.22 | 1.15 | 1.06 | 4.17 | 3.55 | 1.54 | 3.77 | 2.28 | 3.75 | 3.7 | 1.39 | 3.84 | 3.33 | 4.25 | 0.32 | 0.85 | 4.15 | 3.35 | 3.25 | 4.28 | 2.21 |

2-back

| CH | 1 | 2 | 3 | 4 | 5 | 6 | 7 | 8 | 9 | 10 | 11 | 12 | 13 | 14 | 15 | 16 | 17 | 18 | 19 | 20 | 21 | 22 |
|---|---|---|---|---|---|---|---|---|---|---|---|---|---|---|---|---|---|---|---|---|---|---|
| bg | 2.93 | 4.27 | 7.38 | 0.83 | 5 | 2.73 | 6.22 | 5.71 | 5.6 | 0.95 | 0.56 | 7.04 | 5.2 | 3.66 | 2.75 | 0.04 | 3.35 | 5.24 | 7.14 | 7.54 | 1.35 | 7.06 |
| bg2 | 2.07 | 3.02 | 5.22 | 0.59 | 3.53 | 1.93 | 4.4 | 4.03 | 3.96 | 0.67 | 0.4 | 4.98 | 3.67 | 2.59 | 1.95 | 0.03 | 2.37 | 3.7 | 5.05 | 5.33 | 0.95 | 4.99 |
| bg3 | 1.69 | 2.46 | 4.26 | 0.48 | 2.89 | 1.58 | 3.59 | 3.29 | 3.23 | 0.55 | 0.33 | 4.06 | 3 | 2.11 | 1.59 | 0.02 | 1.93 | 3.02 | 4.12 | 4.35 | 0.78 | 4.08 |

## Caption List

**Fig. S1** Four guidelines used for the current Artinis holder and optode configuration.

(A) Rx4 is positioned at AFz, and both Rx4 and Rx5 are aligned along the midline. (B) The lower receiver optode arrays intersect the midline at angles $q_R$ and $q_L$, respectively. (C) The interior angles

formed at Rx4 and Rx5 are fixed at $q_B$ and $q_T$, respectively. (D) The optodes arranged diagonally from Rx4 form an arc.

**Fig. S2** Virtual deformation algorithm for the current Artinis holder.

(A) The reference optode (black dot), Rx4 in this case, is placed on AFz. A reference arc is then drawn connecting AFz and Cz. (B) The distance between Rx4 and Rx5 is determined by calculating the cosines of the fixed angles $q_T$ and $q_B$. The position of Rx5 is then fixed at that distance along the reference arc. (C) The reference arc is rotated based on the fixed angles $q_R$ and $q_B$, and an approximately horizontal arc is drawn. (D) Two points located 30 mm from the two previously determined optode positions (Rx4 and Rx5) are obtained and optodes are placed at these points. (E) Two optodes are placed at points 30 mm from the optode positions determined in (D) and located on the approximately horizontal arc defined in (C). (F) Two diagonal arcs that pass through the optodes determined in (D) and the reference optode Rx4 are drawn. (G) Optodes are placed at points on the arcs determined in (F), located 30 mm from the positions of either the reference optode or the optodes determined in (D). (G) Three optodes are placed at points 30 mm from the positions of either the reference optode or the optodes determined in (D & E) and located on the diagonal arc defined in (F). (H) Two points, each located 30 mm from the optode positions determined in (E & G), are obtained, and optodes are placed at these points (left side is shown). (I) Two optodes are placed at points 30 mm from the optode positions determined in (H) and located on the approximately horizontal arc defined in (C) (left side is shown). (J) Two points, each located 30 mm from the optode positions determined in (E & I), are obtained, and optodes are placed at these points (left side is shown).

**Fig. S3** Estimated optode locations and SDs for actual and virtual registration on the head (left) and cortical (right) surfaces.

Estimated optode locations and SDs for actual and virtual registration on the head (left) and cortical (right) surfaces. Estimated locations from actual and virtual registrations are shown as blue and red circles, respectively, with circle size indicating the SD.

**Fig. S4** Differences in RT (left) and ACC (right) across task conditions.

Differences in RT (left) and ACC (right) across task conditions. ***: $p < .001$. Error bars represent standard errors.

**Fig. S5** Significantly activated CHs for each analysis method for deoxy-Hb in the 2-back condition: NC (top left), SSR (top right), $PCA_{opt}$ (bottom left), and $PCA_{max}$ (bottom right).

Significantly activated CHs for each analysis method for deoxy-Hb: NC (top left), SSR (top right), $PCA_{opt}$ (bottom left), and $PCA_{max}$ (bottom right). Numbers indicate CH numbers. The color of each circle indicates the magnitude of activation, as shown in the color bar.

**Fig. S6** Histogram of maximum CSU values. Red bars indicate oxy-Hb, and blue bars indicate deoxy-Hb. The solid red lines and shaded areas represent the exploration range for oxy-Hb, whereas the dashed blue lines and shaded areas represent the exploration range for deoxy-Hb.

**Fig. S7** Spatial distribution of $BF_{01}$ values for oxy-Hb. The color of each circle indicates the magnitude of BF01 values, as shown in the color bar.

**Fig. S8** Spatial distribution of $BF_{01}$ values for deoxy-Hb. The color of each circle indicates the magnitude of BF01 values, as shown in the color bar.

**Table S1** Results of one-sample t-tests (vs. 0) for channels significantly activated for deoxy-Hb by at least one of the four analysis methods.

*Notes.* P-values are corrected for multiple comparisons using the Bonferroni correction. Cortical region abbreviations: middle frontal gyrus (MFG), inferior frontal gyrus (IFG), superior frontal gyrus (SFG), precentral gyrus (PreCG), left (L), right (R).

**Table S2** Results of the Bayesian t-tests for the 0-back condition (top) and 2-back condition (bottom).

**Table S3** Results of the sensitivity analysis. The first and second rows correspond to oxy-Hb, and the third and fourth rows correspond to deoxy-Hb. Odd-numbered rows represent the 0-back condition, and even-numbered rows represent the 2-back condition.
